# A Trimming Strategy for Mass Defects in Hemispherical Resonators Based on Multi-Harmonic Analysis

**DOI:** 10.3390/mi16040480

**Published:** 2025-04-18

**Authors:** Yimo Chen, Fanrui Kong, Kai Zeng, Xiang Xi, Yan Shi, Dingbang Xiao, Xuezhong Wu

**Affiliations:** College of Intelligence Science, National University of Defense Technology, Changsha 410073, China; chenyimo@nudt.edu.cn (Y.C.); kongfanrui23@nudt.edu.cn (F.K.); xixiang@nudt.edu.cn (X.X.); yanshi@nudt.edu.cn (Y.S.); dingbangxiao@nudt.edu.cn (D.X.); xzwu@nudt.edu.cn (X.W.)

**Keywords:** frequency split, harmonic, hemispherical resonator, ion beam etching, mass defect

## Abstract

This study investigates the impact of etching trimming parameters on the multiple harmonics of the mass distribution in hemispherical resonators and proposes a novel 1st harmonic trimming scheme. As mass balancing technology advances, the extension of identification and trimming from frequency split to multiple harmonics remains a challenge. Initially, a multi-harmonic identification scheme based on spurious mode detection was established, considering the influence of the first three harmonics of the mass distribution on the dynamic characteristics of hemispherical resonators. Finite element method modeling and analysis revealed that common structural geometric errors significantly introduce the 1st harmonic. By integrating a rectangular pulse function into the mass distribution function to simulate etching grooves, spectral analysis revealed that groove depth and width determine the amplitude and gradient of introduced harmonics. This research introduces an innovative discrete trimming scheme aimed at addressing the frequency split and mode mismatch issues associated with traditional single-point trimming of the 1st harmonic. By decomposing the trimming task into primary and auxiliary etching grooves, the 4th harmonic introduced by the primary etching is compensated by the secondary 4th harmonic introduced by the auxiliary etching, achieving decoupling of the 1st harmonic from frequency split during the trimming process. The scheme was verified through finite element simulations and experimental testing. Results demonstrate that, for a similar reduction in the 1st harmonic, the variation in frequency split during the discrete trimming process is only 11% of that observed in single-point trimming, facilitating efficient and low-damage trimming of the 1st harmonic.

## 1. Introduction

Gyroscopes are essential for measuring angular motion in inertial navigation and attitude control systems. Coriolis vibratory gyroscopes (CVGs), in particular, offer significant advantages over traditional gyroscopes, including extended lifespan, low power consumption, and resistance to radiation. These characteristics make CVGs ideal for high-precision applications across various domains, from aerospace to marine systems [1].

Hemispherical resonator gyroscopes (HRGs), which employ a fully symmetric three-dimensional shell design and utilize hemispherical resonators made from high-purity fused quartz as the sensitive elements, operate in a whole-angle mode and have been reported as one of the best-performing CVGs, with widespread applications across various scenarios [2,3]. The manufacturing precision of the hemispherical resonator directly dictates the upper limit of the HRG’s performance. Ideally, the mass distribution of the hemispherical resonator should exhibit isotropic characteristics in the circumferential direction, with the center of mass remaining stationary during the resonance of the *N* = 2 wine-glass modes. However, due to the influence of process-induced errors, the structure often exhibits geometric imperfections, leading to non-uniform mass distribution. The complex non-ideal mass distribution is typically described using Fourier series, which superimposes imbalance terms from various harmonics upon the ideal nominal mass. Among the various harmonics, the first four harmonics of the mass distribution have the most significant impact on the dynamic characteristics of the resonator and the gyroscope performance in the *N* = 2 wine-glass modes.

Previous studies have extensively investigated the impact of the 4th harmonic on frequency split and mode mismatch in *N* = 2 wine-glass modes [4,5,6,7,8,9,10,11,12,13,14,15,16]. This mismatch causes standing wave drift, reducing the gyroscope’s sensitivity and potentially leading to loss of operational capability. Additionally, the requirement for greater orthogonal control forces introduces circuit noise [5,16]. Ion beam etching, laser ablation, and chemical etching are employed as processing techniques for frequency split trimming. Studies by Huo et al. [14], Zhang et al. [15], and Wang et al. [16] using focused ion beam technology have achieved frequency splits below 0.001 Hz. Hamelin et al. introduced laser-induced methods to compensate for manufacturing defects by adjusting local stiffness near support columns [12]. Hu et al. and Zeng et al. analyzed the effects of femtosecond laser trimming on frequency deviation, comparing hole- and groove-trimming methods [17,18]. Luo et al. and Pan et al. enhanced chemical etching efficiency by developing predictive models and controlling etching parameters, achieving frequency splits below 0.05 Hz [19,20]. While these techniques have demonstrated effectiveness in addressing frequency split, the growing demand for higher gyroscope performance necessitates a more comprehensive approach that considers the impact of multiple harmonics.

As research progresses and the performance requirements for gyroscopes escalate, the impact of the first three harmonics on the resonator and gyroscope performance has garnered significant attention from researchers. Firstly, unbalanced mass can cause support loss and reduce the quality factor of resonators. Luo et al. analyzed the support loss in cylindrical resonators with unbalanced mass [21]. Jeanroy et al. [3] and Remillieux and Delhaye [22] developed a mass unbalance model showing that a defect as small as one thousandth can drop the hemispherical resonator’s quality factor from 10 million to about 0.6 million, increasing drift error by approximately 15 times. The 1st harmonic from laser processing deviations significantly impacts anchor loss [23]. Asymmetrical trimming introduces 1st and 2nd harmonics, reducing micro-shell resonators’ quality factor by 10–90% [24]. In addition, unbalanced mass defects in resonators cause deformation, leading to additional forces in the gyroscope’s measurement and control circuits, resulting in output drift [25]. Non-ideal hemispherical resonators can produce parasitic forced vibrations during base excitation, suggesting the need to balance resonator defects [26,27]. These studies collectively indicate that the first three harmonics have numerous deleterious effects on the performance of resonators and gyroscopes; hence, it is imperative to incorporate multi-order harmonics into the mass trimming process in the future, alongside frequency split trimming.

At present, some identification and trimming techniques have involved the first three harmonics of the mass distribution. Basarab et al. investigated resonator thickness variations and developed a magnetic sensor device to detect unbalanced mass parameters in metallic cylindrical shells [28,29]. They also proposed a chemical etching-based balancing process [30]. Tao et al. and Liang et al. identified the 2nd harmonic using frequency splits in *N* = 1 modes and *Z*-axis vibrations [31,32]. Ning et al. analyzed standing wave precession rates to locate unbalanced mass and improved resonator performance by removing defective mass in harmonic form [33]. A method to identify eccentric mass in hemispherical resonators using ion beam etching was proposed, effectively suppressing the 1st and 3rd harmonics [34]. Previous studies have predominantly focused on the trimming of frequency split (the 4th harmonic of the mass distribution) or other specific harmonics, yet there has been no systematic analysis of the interactions between multiple harmonics and the comprehensive impact laws of the trimming process. Notably, Sagem disclosed a mass balancing technique that characterized the unbalanced mass in terms of parameters of 6 degrees of freedom and frequency split in 2014. After a multi-step balancing process, the parameters in the 6 degrees of freedom were iteratively refined from 800 ppm to within 10 ppm, and the frequency split was diminished to 0.5 mHz. However, the underlying mechanism and operational details of the technique remained undisclosed [3,22]. With the advancement of gyroscopic performance and mass trimming techniques, the demand for trimming has expanded from the trimming of the frequency split to the multiple harmonic trimming within mass defects. However, the integrated impact patterns of etching and other trimming process parameters on multiple harmonics remain unclear, hindering the efficient implementation of comprehensive multi-harmonic trimming and thus constraining the development of mass balancing technologies.

By establishing a trimming parameter model that links the mass distribution function with the harmonic spectrum, this paper analyzes the impact of these trimming parameters. Furthermore, a discrete trimming scheme is proposed to compensate for frequency split arising during the trimming process of the first harmonic. The content of this paper is as follows: Section II analyzes the inertial force effects of the first three harmonics based on the mass ring model and identifies these harmonics using this effect. Simulations of the first three harmonics induced by structural errors are conducted. Section III establishes a trimming model to analyze the influence of process parameters on harmonics. Section IV examines the discrete trimming scheme, simulates it using the finite element method, and validates it through experiments.

## 2. Harmonic Identification and Structural Error

### 2.1. Dynamic Model of the Mass Ring

**Figure 1:**
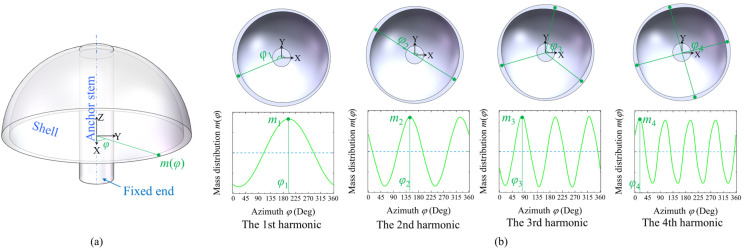
Model of an imperfect hemispherical resonator. (**a**) Schematic diagram of the resonator. (**b**) The first four harmonics of the mass distribution.

The structure of a hemispherical resonator is composed of a shell that generates standing wave vibrations and an anchor stem used for support and fixation to the carrier, as depicted in Figure 1. This anchor stem can be conceptualized as a cantilever beam that is fixed at one end and carries mass and load at the other. In an ideal hemispherical resonator, the vibrating mass should be uniformly distributed circumferentially around the shell, ensuring that the mass at any azimuthal position is identical, thereby maintaining the balance of the center of mass during resonance. However, due to the influence of manufacturing or material defects, the mass distribution is often non-uniform. To describe the complex array of mass defects, a mass ring model is established, wherein the mass distribution along the circumference is considered a continuous signal with a period of 2π. This continuous signal is expanded in the form of a Fourier series:

(1) $$m(\varphi )=m_{0}+\sum_{i=1}^{\infty }m_{i}\cos i(\varphi -\varphi_{i}),$$

where *m*(*φ*) is the mass at azimuth angle *φ*, *m*_0_ is the nominal mass, and *m_i_*, *φ_i_* represent the *i*-th harmonic component and azimuth of the mass distribution, respectively.

Let the symmetric axis of the hemispherical resonator coincide with the *Z*-axis. According to previous work [34], when the hemispherical resonator operates in the *N* = 2 wine-glass modes, the 1st and 3rd harmonics of the unbalanced mass generate eccentric inertial forces *F_B_*(*t*) along the radial direction:

(2) $$F_{B}(t)=-\frac{\pi }{2}\omega^{2}A\sin(\omega t+\phi )[m_{1}\cos(\varphi_{1}-\theta )+m_{3}\cos3(\varphi_{3}-\theta )],$$

where *ω*, *A*, *ϕ*, and *θ* represent the resonant frequency, amplitude, standing wave phase, and the circumferential azimuthal angle of the hemispherical resonator, respectively, while *t* is the time variable.

The anchor stem is secured at one extremity, while the opposite end is subjected to the influence of an eccentric force *F_B_*(*t*), which induces vibration. The resulting bending deflection of the anchor stem due to this compelled vibration is as follows:

(3) $$B(t)=-A\sin(\omega t+\phi_{c})[m_{1}\cos(\varphi_{1}-\theta )+m_{3}\cos3(\varphi_{3}-\theta )]\cdot U,$$

where the constant coefficient U is a function of the resonator’s geometric dimensions and material attributes. Typically, the operating frequency of a resonator is synchronized with its natural frequency, allowing the quadratic term of the angular frequency *ω*^2^ to be integrated into the constant coefficient U.

The 2nd harmonic, in the *N* = 2 wine-glass modes, generates an axial inertial force along the *Z*-axis:

(4) $$F_{Z}(t)=-\pi \omega^{2}A_{Z}m_{2}\cos2\varphi_{2}\cos(\omega t+\phi ),$$

where *A_Z_* is the axial component, along the *Z*-axis, of the resonant amplitude. The anchor stem is affixed at one end, while the opposing end experiences an axial force *F_Z_*(*t*) that compels it to vibrate along the axis. The ensuing axial vibration can be described as follows:

(5) $$Z(t)=-A_{Z}\cos(\omega t+\phi_{c})m_{2}\cos2(\varphi_{2}-\theta )\cdot W.$$

The coefficient *W* in the equation is influenced by the geometric characteristics and material specifications inherent to the anchor stem.

**Figure 2:**
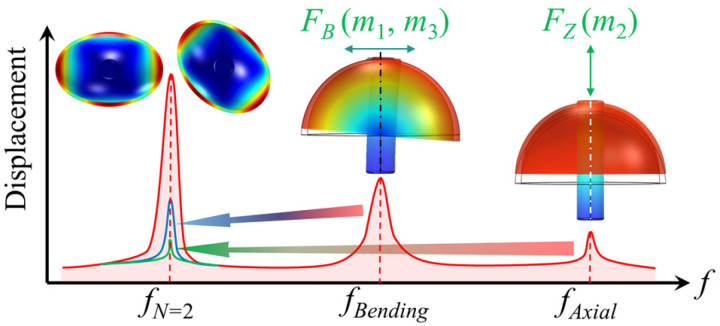
The first three harmonics of the mass distribution induce spurious modes, including bending vibration (blue line and arrow) and axial vibration (green line and arrow), which are coupled into the operating modes.

The radial eccentric forces generated by the 1st and 3rd harmonics of the mass distribution induce bending vibrations in the anchor stem. Concurrently, the 2nd harmonic induces axial forces and vibrations at the same frequency as the resonant standing wave, as illustrated in Figure 2. These two types of vibrations, acting as spurious modes, couple into the operating *N* = 2 wine-glass modes of the hemispherical resonator, leading to increased anchor loss in the resonator and adversely affecting the measurement and control of the gyroscope. Therefore, it is necessary to identify the harmonic information within the mass defects and to trim them to suppress their effects.

### 2.2. Identification of the Harmonics

Due to the generation of two types of inertial forces by the 1st and 3rd harmonics of the mass distribution, which excite bending and axial modes, it is possible to infer the harmonics by measuring the bending vibrations and axial vibrations of the anchor stem. For the 1st and 3rd harmonics, there are four respective amplitudes and azimuthal parameters, *m*_1_, *φ*_1_, *m*_3_, and *φ*_3_, that need to be solved. By exciting standing waves at four different azimuths and measuring the corresponding bending vibrations of the anchor stem at these azimuths, a system of four bending vibration equations can be established to solve for the four parameters.

(6) $$B_{n}=-A_{n}[m_{1}\cos(\varphi_{1}-\theta_{n})+m_{3}\cos3(\varphi_{3}-\theta_{n})]\cdot U.$$

In (6), *B_n_* represents the amplitude of the bending vibration of the anchor stem when the standing wave is aligned along the azimuth *θ_n_*, where *n* can take the values 1, 2, 3, and 4 corresponding to four distinct orientations. Meanwhile, *A_n_* denotes the radial components of the overall vibration amplitudes of the hemispherical resonator at the respective azimuths.

Similarly, by driving the standing wave to two different azimuths, two axial mode equations can be constructed to calculate the amplitude and azimuth of the 2nd harmonic:

(7) $$Z_{n}=-A_{Zn}m_{2}\cos2(\varphi_{2}-\theta_{n})\cdot W,$$

where *Z_n_* represents the axial vibration amplitude of the anchor stem when the standing wave is aligned along the azimuths *θ*_1_ and *θ*_2_, where *n* is 1 or 2 corresponding to two distinct orientations. *A_Zn_* denotes the axial components of the resonator’s amplitudes at these respective azimuthal positions.

According to Equations (6) and (7), the unbalanced mass parameters *m*_1_, *m*_2_, and *m*_3_ are solved by measuring *B_n_*, *Z_n_*, *A_n_*, and *A_Zn_*. The identified harmonic amplitudes, represented by the dimensionless values *B_n_*/*A_n_* and *Z_n_*/*A_Zn_*, are presented in ppm for simplicity.

### 2.3. Structural Errors in Resonators

**Figure 3:**
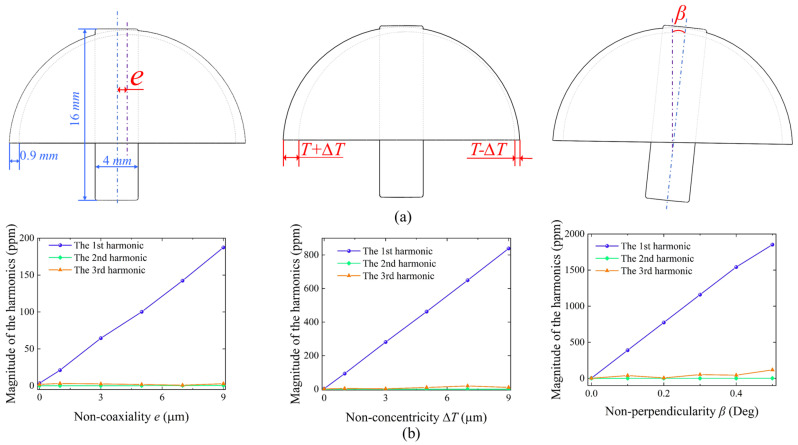
Geometric structural errors induce the first three harmonics of the mass distribution. (**a**) Schematic diagrams of various structural errors. (**b**) Variation of the first three harmonics with structural errors.

Initially, the harmonics of the mass distribution induced by common geometric structural errors in the hemispherical resonator are assessed using the identification methods for the first three harmonics. These errors encompass non-coaxiality *e* between the shell and the anchor stem, non-concentricity ∆*T* of the inner and outer circles of the shell, and non-perpendicularity *β* between the shell and the anchor stem, as illustrated in Figure 3a. Geometric models incorporating various structural errors are first established, and the harmonic components arising from these errors are evaluated using the finite element method. After importing the geometric models into the finite element simulation software, the material is set to silica glass, the base end of the anchor stem is constrained as fixed, and swept meshes are generated. The models are first run to compute the natural frequencies of the *N* = 2 wine-glass modes, followed by a frequency domain calculation where the resonator is excited into two degenerate states. From the dataset of the finite element calculations, vibration data at the top of the anchor stem and at various points on the rim of the shell are extracted, and the harmonics are calculated using the harmonic identification formulas for each order.

The identification results are shown in Figure 3b. Comparing the first three harmonics of the mass distribution, the 1st harmonic increases almost proportionally with the enlargement of geometric errors, with a rate of increase significantly greater than that of the 2nd and 3rd harmonics. Consequently, structural errors such as coaxiality, concentricity and perpendicularity often result in a notably pronounced initial 1st harmonic in the manufactured hemispherical resonators. Although enhancing the precision of mechanical processing can somewhat reduce the unbalanced mass resulting from structural errors, the necessity of trimming harmonics as a subsequent post-processing step is beneficial for improving the uniformity of the resonator’s mass distribution and enhancing the performance potential of the processed hemispherical resonators.

## 3. Process Parameters in Harmonic Trimming

### 3.1. Trimming Parameter Model

After identifying the various harmonics associated with unbalanced mass, the distribution of mass along the circumferential direction of the resonator can be altered by removing or adding material, thereby achieving harmonic trimming and suppressing its adverse impact on the performance of the resonator. A trimming parameter model based on the circumferential mass distribution function is established, and the mass distribution function is expanded into a spectral form using Fourier series, describing the influence of trimming parameters on each harmonic order, which aids in devising trimming strategies.

**Figure 4:**
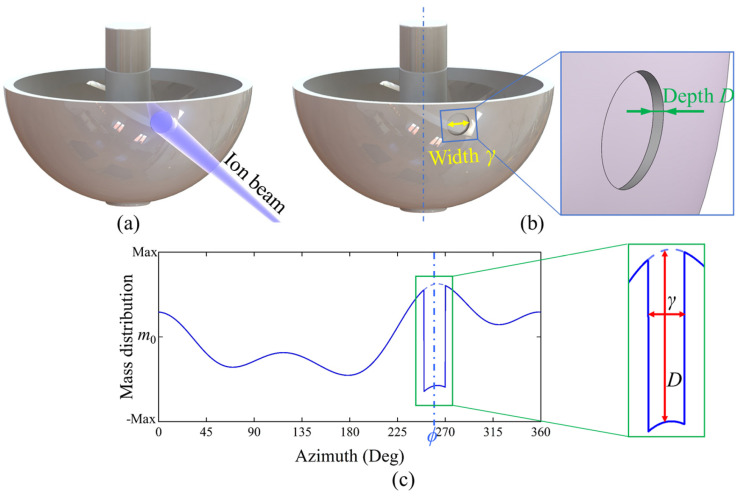
Hemispherical resonator surface trimming model. (**a**) Schematic diagram of ion beam etching. (**b**) Parameters of the etching groove. (**c**) Mass distribution function after trimming.

When an ion beam is continuously directed at a specific azimuthal position on the surface of the resonator, as shown in Figure 4, to form an etching groove and remove a portion of the material from the resonator’s surface, thereby reducing the mass in that area, a discontinuity in the circumferential mass distribution of the resonator is produced. This discontinuity can be described using a rectangular pulse function *δ*(*φ*):

(8) $$\delta =\left\{\begin{array}{c} \begin{array}{cc} D\; & \varphi \in [\phi -\frac{\gamma }{2},\phi +\frac{\gamma }{2}] \end{array}\\ \begin{array}{cc} 0\; & \varphi \notin [\phi -\frac{\gamma }{2},\phi +\frac{\gamma }{2}] \end{array} \end{array}\right.,$$

where *D* represents the amplitude height of the rectangular pulse, characterizing the radial depth of the etching groove along the mass ring. *ϕ* denotes the azimuthal angle of the center of the etching groove. *γ* is the width of the rectangular pulse, ranging from 0 to 2π, which characterizes the aperture or width of the etching groove. In this study, cylindrical approximations for parameters *D* and *r* are employed to simplify the geometric modeling of etching grooves. This assumption is valid only for very small structures, where the curvature of the hemispherical resonator’s surface can be neglected. For larger structures, the cylindrical approximation may introduce significant errors, and more complex geometric models should be considered. The applicability of this assumption is explicitly clarified in the parameter definition section.

**Figure 5:**
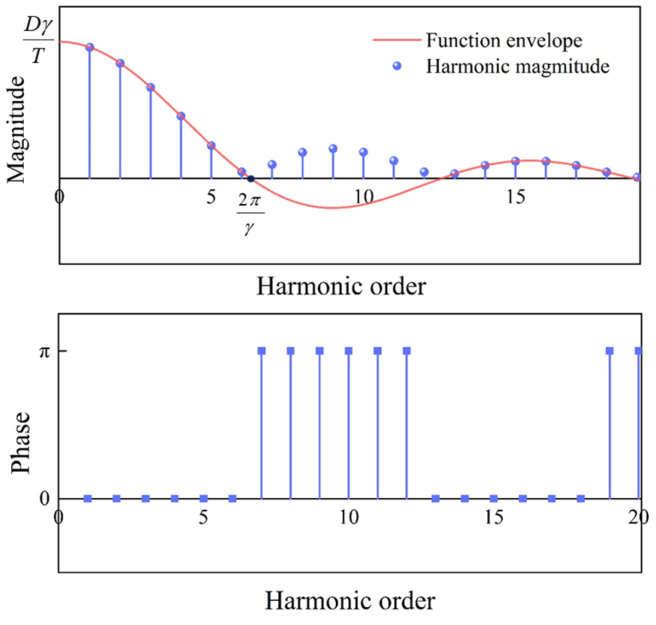
Frequency spectrum of the rectangular pulse function *δ*(*φ*).

Taking the center azimuth of a single etching groove as the origin and with a period *T* of 2π, the rectangular pulse function *δ*(*φ*) can be expanded using a Fourier series:

(9) $$F_{n}=\frac{1}{T}\int_{-\frac{T}{2}}^{\frac{T}{2}}\delta (\varphi )e^{-jn\omega_{1}\varphi }d\varphi =\frac{1}{T}\int_{-\frac{\gamma }{2}}^{\frac{\gamma }{2}}De^{-jn\omega_{1}\varphi }d\varphi =\frac{D\gamma \sin\frac{n\omega_{1}\gamma }{2}\gamma }{T\frac{n\omega_{1}\gamma }{2}}=\frac{D\gamma }{T}\mathrm{Sa}(\frac{n\omega_{1}\gamma }{2}),$$

where $\omega_{1}=\frac{2\pi }{T}=1$, *j* denotes the imaginary unit, and *n* represents the harmonic order. Based on the properties of the sampling function Sa, the frequency spectrum *F_n_* is plotted. Figure 5 illustrates the impact of the trimming parameters *D* and *γ* on the amplitudes of the various harmonics introduced by the etching groove, with *D* and *γ* values of 1 in the figure. The phases of the lower-order harmonics before the intersection of the envelope lines are all zero, meaning that when a rectangular pulse is introduced, its transformation into lower-order harmonics (orders less than 2π/*γ*) is oriented in the direction of the center’s azimuth of the etching groove.

**Figure 6:**
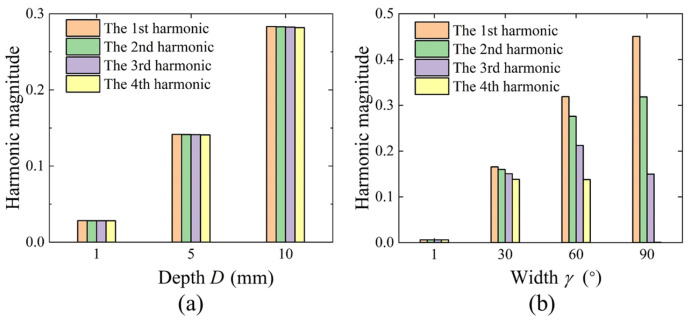
Relationship between the magnitudes of the first four harmonics and the trimming parameters *D* and *γ*. (**a**) When *γ* is set to 10°, the harmonic magnitudes vary with depth *D*. (**b**) When *D* is set to 1 mm, the harmonic magnitudes change with width *γ*.

According to the spectral expressions and Figure 5, with the width *γ* determined, the amplitudes of the harmonics corresponding to the rectangular pulse function are directly proportional to the depth of the etching groove. As shown in Figure 6, with a fixed width *γ* of 10 degrees, the first four harmonics increase concurrently with the increase in depth *D*. With a constant depth of a single etching groove (*D* = 1), the width *γ* affects the amplitude of the 1st harmonic, with an approximate linear relationship. This indicates that as the width of the etching groove increases, a smaller depth can achieve the targeted trimming amount. Additionally, apart from the coefficient term affecting the amplitude of the harmonics, the width *γ* also influences the gradient of the harmonics introduced by the etching groove. In Figure 5, the envelope of the Sa function decreases progressively from the 1st harmonic to the first intersection with the coordinate zero axis at 2*π/γ*. The smaller the width *γ*, the larger the abscissa of the intersection point of the envelope line with the zero axis and the closer the first four harmonics are to each other. As *γ* increases, the intersection position 2*π/γ* of the envelope line and the coordinate axis decreases, the gradient increases, and the amplitudes of the first few harmonics decay progressively with each order. When *γ* reaches 90 degrees, it almost does not introduce a 4th harmonic.

### 3.2. Simulation Verification

**Figure 7:**
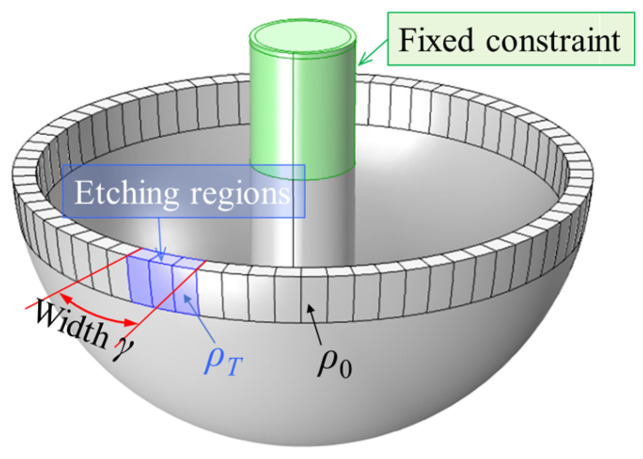
Schematic diagram of simulation model for trimming of hemispherical resonator.

To verify the rectangular pulse parameter model for trimming, a geometric model and simulation procedure are established to simulate the effects of the trimming parameters *D* and *γ* on the trimming process. Initially, an ideal hemispherical resonator model is constructed, with its shell rim section evenly divided into 72 segments along the circumferential direction as in Figure 7 and imported into finite element analysis software. The model is initialized with silica glass material, characterized by a material density *ρ*_0_ of 2203 kg/m³. A number of these segments on the shell rim are designated as the simulated etching regions, and the material density of these areas is assigned as follows:

(10) $$\rho_{T}=\rho_{0}-k\rho_{0},$$

where *k* represents the depth coefficient, which is equivalent to the depth parameter *D* in the trimming model. The value of *k* and the coverage area of the simulated etching region are used to emulate the depth *D* and width *γ* of the rectangular pulse, respectively.

**Figure 8:**
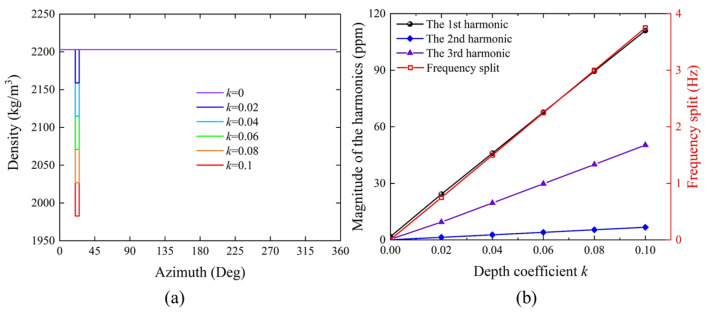
Impact of simulated etching depth on harmonic distortions. (**a**) Density distribution along the circumferential direction of the model under various depth coefficients. (**b**) Identification of the first three harmonics and frequency split from simulation results at varying depth coefficients.

Initially, the sector spanning 20 to 25 degrees is designated as the simulated etching region. With an etching width of 5 degrees, an increase in the depth coefficient results in a corresponding decrease in the density of the etching region, thereby simulating the state of material removal and mass reduction. The density distribution at different depth coefficients *k* is shown in Figure 8a, simulated for different depths *D* of the rectangular pulse model. The simulation program is then executed to calculate the natural frequencies of the *N* = 2 wine-glass modes, from which the frequency split is determined. Subsequently, frequency domain calculations for two degenerate modes are conducted, and data from the top of the anchor stem and the shell rim are extracted and substituted into the harmonic identification formulas to compute the harmonics of each order. The simulation results, as depicted in Figure 8b, show that the identified first three harmonics and frequency split linearly increase with the augmentation of the depth coefficient *k*, a pattern consistent with the variation in density.

**Figure 9:**
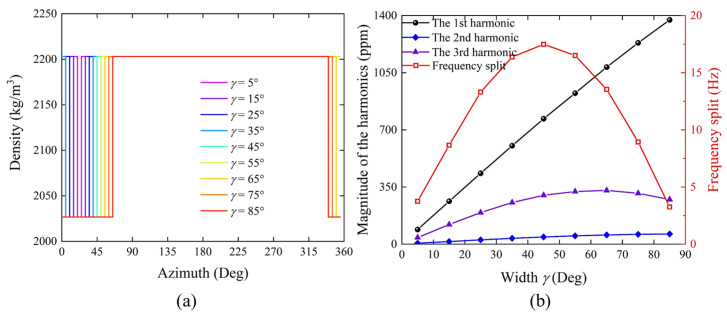
Influence of etching region width on harmonic distortions. (**a**) Density distribution along the circumferential direction of the model for various widths of the etching region. (**b**) Identification of the first three harmonics and frequency split from simulation results corresponding to varying widths of the etching region.

With a depth coefficient *k* of 0.008, the trimming density distribution for different etching region widths is compared in Figure 9a, simulated for different widths *γ* of the rectangular pulse function model. After running the simulation, the frequency split and each harmonic order are calculated using the same procedure. The results in Figure 9b indicate that as the trimming width increases, the 1st harmonic essentially increases proportionally, and the gradient of the resulting harmonics also increases accordingly. The identified 1st harmonic increases almost linearly with the increase in the width of the etching regions; however, due to the increase in harmonic gradient, the 2nd and 3rd harmonics and the frequency split no longer vary linearly with the width. As the width of the etching regions exceed 45 degrees, the introduced frequency split gradually decreases. The finite element simulation results validate the trimming parameter model based on the mass distribution function and spectral analysis.

## 4. Discrete Trimming Scheme

### 4.1. Frequency Split Compensation

Compared to mechanical grinding, laser ablation, and chemical etching, ion beam etching is a non-contact processing technology with atomic-level material removal capability and precise localization of micro-defects. By controlling energy density and incident angle, issues such as thermal stress, thermoelastic losses, mechanical damage, and surface contamination can be minimized, thereby reducing the impact on the quality factor [14,15,16]. Ion beam etching is typically employed with a small diameter to ensure that the energy is concentrated on the target area. The diameter of a single etching groove produced is often within 3 mm, which corresponds to a width of less than π/19 (approximately 20 degrees) in a 20 mm diameter hemispherical resonator. As per the numerical analysis and simulation results mentioned earlier, under these conditions, the gradient of the harmonics introduced by the etching groove is relatively small. Therefore, single-point trimming (SPT) of the 1st harmonic also introduces higher-order harmonics, including the 4th harmonic. Various geometric structural errors necessitate a larger amount of trimming for the 1st harmonic, which consequently increases the frequency split of the *N* = 2 wine-glass modes due to the 4th harmonic. Mode mismatch prevents the resonator from functioning effectively as a sensitive element in a gyroscope.

A discrete trimming (DT) method has been proposed to circumvent the mode mismatch issues associated with SPT. This method is based on a single-point etching groove, which serves as the primary etching (PE) groove corresponding to the 1st harmonic, with a trimming amount denoted as *T*_1_. On the basis of the PE groove, auxiliary etching (AE) grooves of the same width are added at positions 45 degrees to the left and right, with trimming amounts dependent on the etching depth, each being half of the PE groove’s trimming amount, i.e., *T*_1_/2, to compensate for the 4th harmonic introduced by the SPT. The principle of DT method is based on the fact that the PE groove at the center introduces a 4th harmonic with its peak phase aligned with the center azimuth; meanwhile, the positions 45 degrees to the left and right of the center are both trough positions for the introduced 4th harmonic. By introducing secondary 4th harmonics at the trough positions with AE grooves, the phase of these secondary 4th harmonics is opposite to that of the original 4th harmonic, and the combined trimming effect of the two AE grooves cancels out the trimming effect of the central PE groove, achieving a compensatory effect. The total contributions of the 1st harmonic *T*_1_′ and 4th harmonic *T*_4_′ introduced by the three etching grooves are as follows:

(11) $${T_{1}}^{\prime }=T_{1}+2\cos\left(45{}^{\circ}\right)\frac{T_{1}}{2}=\left(1+\frac{\sqrt{2}}{2}\right)T_{1},$$

(12) $${T_{4}}^{\prime }=T_{4}+2\cos\left(4\times 45{}^{\circ}\right)\frac{T_{4}}{2}=0.$$

According to Equation (11), the trimming amount of the central PE groove is approximately 0.6 times that required by the SPT method. This helps to mitigate the potential destructive effects on the local structure of the hemispherical resonator that may arise from excessive trimming amounts.

**Figure 10:**
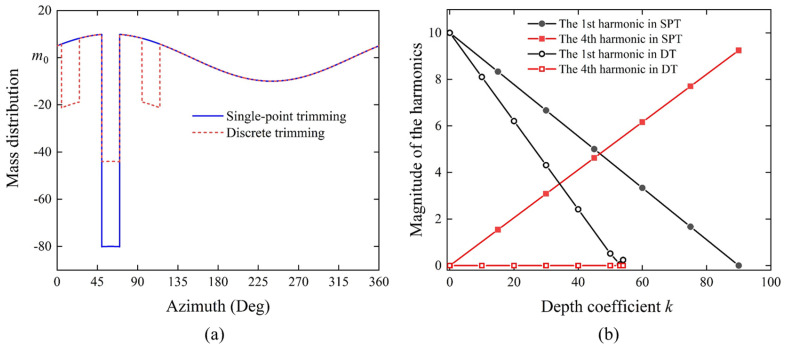
Comparison of single point trimming (SPT) and discrete trimming (DT) methods. (**a**) Comparison of mass distribution functions. (**b**) Variation of 1st and 4th harmonic amplitude values with depth coefficient k in both schemes.

As shown in the mass distribution function depicted in Figure 10a, the initial amplitude of the 1st harmonic is 10, with an orientation of 60 degrees, and all other harmonics are 0. When employing the SPT method, an etching groove with a depth of 90 and a width of 20 degrees at the 60-degree position is required to reduce the amplitude of the 1st harmonic to 0; however, this also introduces a 4th harmonic with an amplitude of 9.245 as shown in Figure 10b. In contrast, when using the DT method, three etching grooves each with a width of 20 degrees are introduced: a central PE groove with a depth of 54, and two AE grooves each with a depth of 27. This approach can reduce the 1st harmonic to 0.2, while the 4th harmonic remains essentially unchanged.

### 4.2. Simulation Results

To validate the DT scheme, the rim region of the ideal hemispherical resonator model is divided into 72 equal segments. The model is imported into finite element software and initialized with silica glass material. Based on the material density *ρ*_0_, the material density values of each segment in the rim region are assigned according to the azimuthal angle, thereby embedding various harmonics into the hemispherical resonator model:

(13) $$\rho (\varphi )=\rho_{0}\left(1+\sum_{i=1}^{4}\rho_{i}\cos i(\varphi -\varphi_{i})\right).$$

**Figure 11:**
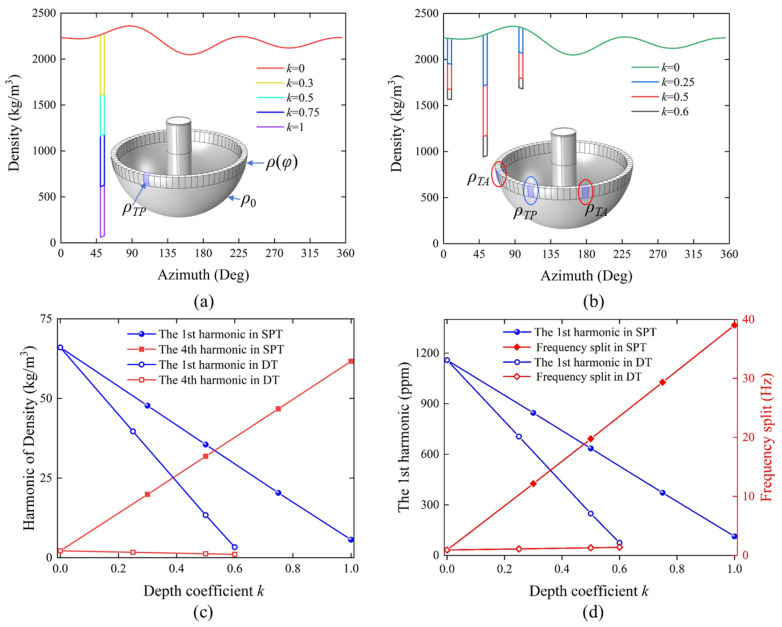
Finite element simulation of SPT and DT. (**a**) Circumferential density distribution function of the resonator model in the SPT scheme. (**b**) Circumferential density distribution function of the resonator model in the DT scheme. (**c**) Variation of harmonics derived from density distribution for both schemes with respect to the depth coefficient *k*. (**d**) Variation of the identified 1st harmonic and frequency split for both schemes with respect to the depth coefficient *k*.

The 1st and 4th harmonics of the density distribution are initialized with the following parameters: *ρ*_1_ is set to 0.03 and *φ*_1_ is set to 50 degrees, while *ρ*_4_ is set to 0.001 and *φ*_4_ to 0 degrees. Both the SPT and DT schemes are simulated to trim the 1st harmonic in the model. As shown in Figure 11a,b, the segment corresponding to the orientation to be trimmed is designated as a single-point etching region, which also serves as the PE region in the DT scheme, with a width of 5 degrees and a trimming amount equal to the product of the depth coefficient *k* and initial density *ρ*_0_. Building upon the previously embedded harmonic density values *ρ*(*φ*), the density of the PE region is modified to the following:

(14) $$\rho_{TP}(\varphi )=\rho (\varphi )-k\rho_{0}.$$

In the DT method, additional regions located 45 degrees to either side are designated as PE regions, each with a width of 5 degrees. The density values assigned to these areas are as follows:

(15) $$\rho_{TA}(\varphi )=\rho (\varphi )-\frac{k\rho_{0}}{2}.$$

As depicted in Figure 11c, the 1st and 4th harmonics in the density distribution are compared between the SPT and DT methods as a function of the depth coefficient *k*. By employing the SPT method and increasing the depth coefficient *k* of the trimming region to 1, the 1st harmonic in the density distribution is reduced from 66 kg/m^3^ to 5.7 kg/m^3^, a decrease by an order of magnitude. However, concurrently, the 4th harmonic increases from 2.2 kg/m^3^ to 61.7 kg/m^3^. When the depth coefficient *k* reaches 1, the density in the trimming region is reduced to near zero, indicating that the material is almost completely removed. In practical operations, this would significantly compromise the structural integrity. In contrast, the DT method sets the depth coefficient *k* of the PE region to 0.6, with the depth coefficients of AE regions on either side set to 0.3, which can reduce the 1st harmonic in the density distribution by an order of magnitude to 3.3 kg/m^3^, while the 4th harmonic remains essentially unchanged.

Running the finite element simulation program initially calculates the natural frequencies to obtain the frequency split of the two *N* = 2 wine-glass modes; subsequently, the frequency domain calculation is performed. The results, which include the vibration data from the top of the anchor stem and the rim of the resonator, are substituted into the identification formulas to decompose and extract the 1st harmonic during the simulated etching process.

Figure 11d illustrates that when using the SPT method, as the depth coefficient *k* increases to 1, the 1st harmonic decreases by one order of magnitude from 1158.5 ppm to 112.7 ppm; however, the frequency split increases dramatically by 38.2 Hz. In contrast, when employing the DT method, with the depth coefficient set to 0.6, the 1st harmonic decreases by one order of magnitude to 74.7 ppm, while the frequency split increases by only 1.4 Hz. Comparing the variations in the 1st harmonic and frequency split between the two trimming schemes, the proposed DT method effectively compensates for the frequency split caused by the SPT of the 1st harmonic. Moreover, the required depth of individual etching grooves is smaller, thus contributing to the preservation of the structural and morphological integrity of the resonator.

### 4.3. Experimental Results

**Figure 12:**
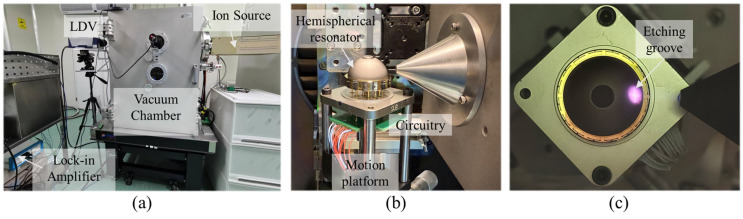
Experimental platform: (**a**) Schematic diagram of the experimental system. (**b**) Assembly of hemispherical resonator samples awaiting trimming. (**c**) Etching the sample surface with an ion beam.

As depicted in Figure 12, an integrated experimental platform for harmonic identification and trimming of hemispherical resonators has been established. This experimental platform primarily comprises the following modules: a motion platform, a Laser Doppler Vibrometer (LDV), a lock-in amplifier, an ion beam system, and a vacuum chamber. The motion platform, which includes a motor, grating, rotary table, translation stage, and elevation stage, facilitates three-axis translational and rotational movements to adjust the position and attitude of the resonator sample. The LDV and lock-in amplifier are utilized for modal testing and vibration data acquisition. The ion beam system enables continuous etching of the resonator’s surface to remove material for harmonic trimming. The controllable atmospheric pressure within the vacuum chamber provides a suitable operating environment for both the resonator and the ion beam system.

After assembly with the measurement and control circuitry, the hemispherical resonator sample to be tested is secured on the motion platform. Initially, the position and attitude of the resonator sample are adjusted using the motion platform to enable the LDV to direct the laser onto the sample for acquiring mechanical vibration signals. Via the electrical interface in the vacuum chamber, the lock-in amplifier outputs excitation signals to the sample’s driving electrodes and receives detection signals from the sample’s sensing electrodes.

Initially, the modal parameters of the sample are obtained using the spectrum analysis method and the decay time method, which include the natural frequencies of the *N* = 2 wine-glass modes and the frequency split. Based on the proposed harmonic identification method, a closed-loop control using the driving and sensing electrodes is employed to maintain constant amplitude resonance of the standing wave at four different azimuthal positions, *θ*_1_ to *θ*_4_. The LDV is used to measure the vibration at the anchor stem of the sample as *B*_1_ to *B*_4_, and also measure the vibration at the rim of the shell, which are used as parameters *A*_1_ to *A*_4_ substituted into (6) to calculate the magnitude and azimuth of the 1st harmonic.

By adjusting the position and angle of the resonator sample using the motion platform, the 1st harmonic and its corresponding azimuth *φ*_1_ are aligned with the ion beam outlet. The ion beam is then activated to continuously bombard the surface of the sample’s shell to achieve trimming.

**Figure 13:**
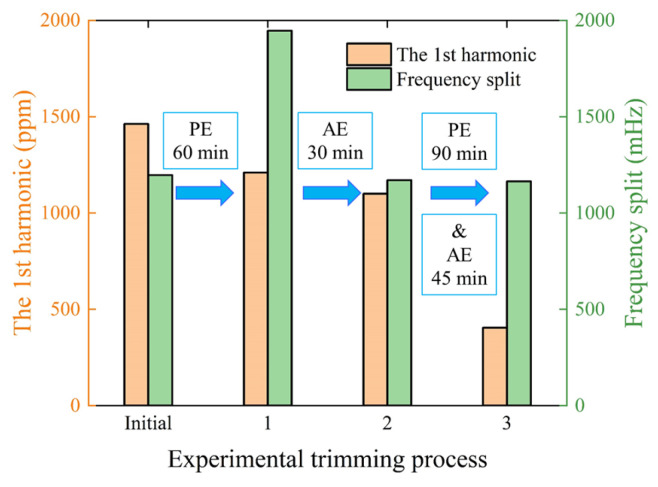
Disassembled experimental results of the DT scheme.

The DT scheme, consisting of PE and AE steps, is first validated on a sample hemispherical resonator, with the results depicted in Figure 13. Following the initial identification of the 1st harmonic and frequency split, a PE groove is created by etching for 60 min at the azimuth *φ*_1_ corresponding to the 1st harmonic. After the primary etching, the 1st harmonic is reduced from 1462 ppm to 1210 ppm, a decrease of 17.2%. However, the frequency split increased by 62.7% from 1196.1 mHz to 1946 mHz. With the ion beam diameter maintained constant, two AE grooves are formed by etching for 30 min on both sides at 45 degrees. This results in a slight reduction of the 1st harmonic from 1210 ppm to 1100 ppm, while the frequency split is reduced from 1946 mHz to 1170 mHz, nearly returning to the initial frequency split. This demonstrates that the AE grooves could suppress frequency split by compensating for the 4th harmonic. Subsequently, a complete DT process is conducted, with the PE groove etched for 90 min and the AE grooves etched for 45 min each. After this process, the 1st harmonic is further reduced to 404.3 ppm, and the frequency split remained almost unchanged at 1164 mHz. This outcome illustrates that the DT approach can effectively reduce the 1st harmonic without introducing the 4th harmonic, thus preventing drastic changes in frequency split.

**Figure 14:**
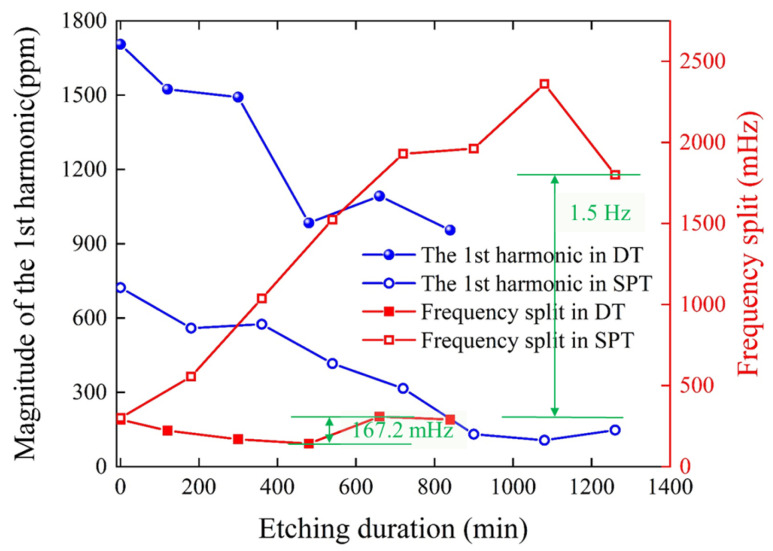
Experimental comparison of the etching process between the SPT and DT schemes.

A DT experiment is also conducted on another resonator sample, and the changes in the 1st harmonic and frequency split during the etching process are compared with the results of the SPT experiment from previous work [34], as shown in Figure 14. In the figure, for DT, each step of trimming includes the total etching time for both the PE and AE grooves. After the SPT process, the 1st harmonic decreases from 723 ppm to 147.6 ppm, a reduction of 575.4 ppm. However, the frequency split sees a sharp increase from 0.3 Hz to 1.8 Hz. In contrast, the DT method, which lasted for 840 min, successfully reduces the 1st harmonic by 750 ppm. Throughout the process, the frequency split exhibits minimal fluctuations, with the maximum deviation reaching only 167.2 mHz, which is approximately 11% of the frequency split variation observed in the SPT scheme. The comparison results demonstrate that DT can achieve decoupling of the 1st and 4th harmonics, which is beneficial for optimizing the mass balance of hemispherical resonators.

## 5. Conclusions

The identification and trimming of multiple harmonics of the mass distribution in hemispherical resonators present greater challenges compared to traditional identification and trimming based on frequency split of resonant modes. Initially, a multi-harmonic identification scheme was employed to analyze the generation mechanisms of these harmonics. A trimming parameter model based on mass distribution functions and spectral analysis was constructed to examine the impact of etching groove parameters on multiple harmonics during the trimming process, which was subsequently validated through finite element simulations.

To address the issue of modal frequency split and mode mismatch caused by traditional single-point trimming of the 1st harmonic, this study proposes a discrete trimming scheme. This scheme compensates for the 4th harmonic introduced by trimming through auxiliary etching grooves, achieving decoupling of the 1st harmonic and frequency split during the trimming process. Finite element simulation results and experimental outcomes demonstrate that this scheme can perform 1st harmonic trimming without exacerbating frequency split.

Future work will explore the optimization of etching process and comprehensive trimming schemes for the first four harmonics, aiming to enhance mass balancing efficiency and improve the surface quality and circumferential symmetry of hemispherical resonators. Additionally, the performance enhancement effects of mass balance on hemispherical resonators and hemispherical resonator gyroscopes will be assessed.

## Data Availability

The original contributions presented in the study are included in the article, further inquiries can be directed to the corresponding author.

## References

[1] Rozelle D.M. The hemispherical resonator gyro: From wineglass to the planets. Proceedings of the AAS/AIAA Space Flight Mechanics Meeting 2009.

[2] Delhaye F. HRG by SAFRAN: The game-changing technology. Proceedings of the 2018 IEEE International Symposium on Inertial Sensors and Systems (INERTIAL).

[3] Jeanroy A., Bouvet A., Remillieux G. (2014). HRG and marine applications. Gyroscopy Navig..

[4] Huo Y., Ren S., Yi G., Wang C. (2020). Motion equations of hemispherical resonator and analysis of frequency split caused by slight mass non-uniformity. Chin. J. Aeronaut..

[5] Xu Z., Zhu W., Yi G., Fan W. (2021). Dynamic modeling and output error analysis of an imperfect hemispherical shell resonator. J. Sound Vib..

[6] Vakhlyarsky D., Sorokin F., Gouskov A., Basarab M., Lunin B. (2022). Approximation method for frequency split calculation of coriolis vibrating gyroscope resonator. J. Sound Vib..

[7] Deng K., Pan Y., Jia Y., Li J., Tang X., Wu W., Yuan J. (2024). Separation of mass and gap errors of hemispherical resonator gyroscopes by varying bias voltage. Meas. J. Int. Meas. Confed..

[8] Senkal D., Ahamed M.J., Trusov A.A., Shkel A.M. (2014). Achieving sub-hz frequency symmetry in micro-glassblown wineglass resonators. J. Microelectromechanical Syst..

[9] Sorokin F., Vakhlyarsky D., Gouskov A. (2022). High rise of ring resonator frequency split due to combination of two harmonics of density defect. Appl. Math. Model..

[10] Wang Y., Asadian M.H., Shkel A.M. (2018). Compensation of frequency split by directional lapping in fused quartz micro wineglass resonators. J. Micromechanics Microengineering.

[11] Ayazi F., Hamelin B., Tavassoli V. (2014). Localized eutectic trimming of polysilicon microhemispherical resonating gyroscopes. IEEE Sens. J..

[12] Choi S.-Y., Kim J.-H. (2011). Natural frequency split estimation for inextensional vibration of imperfect hemispherical shell. J. Sound Vib..

[13] Kim J., Kim J. (2017). Trimming of imperfect hemispherical shell including point mass distributions. Int. J. Mech. Sci..

[14] Huo Y., Wei Z., Ren S., Yi G. (2023). High precision mass balancing method for the fourth harmonic of mass defect of fused quartz hemispherical resonator based on ion beam etching process. IEEE Trans. Ind. Electron..

[15] Zhang W., Zhang Y., Gu H., Lin Z., Wei Q., Zhou B., Zhang R. (2023). The high-precision balance method for hemispherical resonator based on synchronous trimming system. IEEE Sens. J..

[16] Wang C., Ning Y., Huo Y., Yuan L., Cheng W., Tian Z. (2024). Frequency split of hemispherical resonators trimmed with focused ion beams. Int. J. Mech. Sci..

[17] Hu Y., Zhou Y., Zhong H., Zeng K., Sun X., Duan J.A. (2019). Precise dynamic mass-stiffness balancing of cylindrical shell vibrating gyroscope along working modal axis. IEEE Sens. J..

[18] Zeng K., Hu Y., Deng G., Sun X., Su W., Lu Y., Duan J.A. (2017). Investigation on eigenfrequency of a cylindrical shell resonator under resonator-top trimming methods. Sensors.

[19] Luo Y., Gebrel I., Bognash M., Pan Y., Liu F., Asokanthan S., Luo H., Qu T. (2020). Dynamic response and frequency split predictions for cylindrical fused silica resonators. IEEE Sens. J..

[20] Pan Y., Pan Y., Jin S., Jia Y., Yang K., Luo H. (2019). Trimming of imperfect cylindrical fused silica resonators by chemical etching. Sensors.

[21] Luo H., Tao Y., Zeng L., Tang X., Yang K., Luo H. Investigation on the optimal fixation condition of cylindrical resonators. Proceedings of the 28th Saint Petersburg International Conference on Integrated Navigation Systems.

[22] Remillieux G., Delhaye F. Sagem Coriolis vibrating gyros: A vision realized. Proceedings of the 2014 DGON Inertial Sensors and Systems (ISS).

[23] Shi Y., Xi X., Li B., Chen Y., Wu Y., Xiao D., Wu X., Lu K. (2021). Micro Hemispherical Resonator Gyroscope With Teeth-Like Tines. IEEE Sens. J..

[24] Lu K., Xi X., Xiao D., Shi Y., Zhuo M., Wu X., Wu Y. (2019). A study on the trimming effects on the quality factor of micro-shell resonators vibrating in wineglass modes. Micromachines.

[25] Zhbanov Y.K., Zhuravlev V.P. (2007). Effect of movability of the resonator center on the operation of a hemispherical resonator gyro. Mech. Solids.

[26] Pavlovskii A.M., Sarapulov C.A. (1991). Dynamics of a nonideal hemispherical resonator with translational vibration of the base. Int. Appl. Mech..

[27] Huo Y., Ren S., Wei Z., Yi G. (2020). Standing wave binding of hemispherical resonator containing first-third harmonics of mass imperfection under linear vibration excitation. Sensors.

[28] Basarab M.A., Matveev V.A., Lunin B.S., Fetisov S.V. (2017). Influence of nonuniform thickness of hemispherical resonator gyro shell on its unbalance parameters. Gyroscopy Navig..

[29] Basarab M.A., Chumankin E.A., Lunin B.S. Application of a magnetic sensor for determining the mass imbalance of the Coriolis vibratory gyroscope with cylindrical metallic resonator. Proceedings of the 2017 Progress in Electromagnetics Research Symposium—SPRING (PIERS).

[30] Basarab M.A., Lunin B.S., Matveev V.A., Chumankin E.A. (2015). Balancing of hemispherical resonator gyros by chemical etching. Gyroscopy Navig..

[31] Tao Y., Pan Y., Liu J., Jia Y., Yang K., Luo H. (2021). A novel method for estimating and balancing the second harmonic error of cylindrical fused silica resonators. Micromachines.

[32] Liang C., Yang K., Pan Y., Tao Y., Li J., Jin S., Luo H. (2022). Simulations and experiments on the vibrational characteristics of cylindrical resonators with first three harmonic errors. Micromachines.

[33] Ning Y., Wang C., Cheng W., Tian Z. (2024). Anchor loss improvement in hemispherical resonators with ion beams. Int. J. Mech. Sci..

[34] Chen Y., Zeng K., Xi X., Lu K., Shi Y., Xiao D., Wu X. (2024). Identification and trimming of the eccentric mass in shell resonators. Int. J. Mech. Sci..

